# Molecular Profiling and Pathological Evaluation of Bovine Papillomavirus-1 in Cattle in Al-Sharkia, Egypt

**DOI:** 10.1155/vmi/9808789

**Published:** 2025-05-08

**Authors:** Ayman Ahmed Shehata, Elshaima Mohamed Fawzi, Mahran Mohamed Abd El-Emam, Shimaa M. Abdullah, Wafaa Hassan, Asmaa lbrahim Abdelaziz Zin Eldin, Hend E. M. Elsheikh

**Affiliations:** ^1^Department of Animal Medicine, Infectious Diseases, Faculty of Veterinary Medicine, Zagazig University, Zagazig 44511, Egypt; ^2^Department of Biochemistry and Molecular Biology, Faculty of Veterinary Medicine, Zagazig University, Zagazig 44511, Egypt; ^3^Department of Animal Medicine, Internal Medicine, Faculty of Veterinary Medicine, Zagazig University, Zagazig 44511, Egypt; ^4^Department of Microbiology and Immunology, Veterinary Research Institute, National Research Centre, Giza, Egypt

**Keywords:** bovine papillomavirus-1, cattle, histopathology, phylogenetic analysis, sequence

## Abstract

Bovine papillomatosis virus (BPV) is a prevalent cutaneous oncogenic viral disease in cattle, causing economic losses due to reduced milk production, poor carcass quality, and hide damage. Despite BPV's economic significance, molecular information on current strains, genetic relationships, and origins in Egypt is limited, with most studies focusing on electron microscopy and histopathological analysis. The study aimed to genetically analyze BPV-1 circulation in Al-Sharkia, Egypt, and characterize viral strains compared with local and global papillomaviruses isolates. A total of 27 crossbred cattle with clinical symptoms of papillomatosis, such as wart-like lesions on various body parts, were examined. The collected tissue samples underwent histological analysis, revealing typical benign neoplasms such as hyperkeratosis and koilocytes. Polymerase chain reaction (PCR) confirmed the presence of BPV-1 in all samples, with partial amplification of the L1 gene. Sequencing and phylogenetic analysis of three representative samples indicated high similarity to BPV-1 strains from Egypt, Iraq, Turkey, and Belgium, suggesting livestock trading may play a role in disease transmission. The isolates were found to be linked to equine Delta papillomavirus 4 (DPV4) strains, indicating cross-species transmission between cattle and equines. The study marks one of the first reports of BPV-1 infection in cattle in Al-Sharkia, providing crucial molecular insights into Egypt's circulating strains and emphasizing the need for stronger biosecurity protocols in animal trading.

## 1. Introduction

Bovine papillomaviruses (BPVs) are common skin-associated viruses in cattle, belonging to the Papillomaviridae family and characterized by their double-stranded DNA genome. Five genera of Papillomaviruses, including *Delta-*, *Xi-*, *Epsilon-*, *Dyoxi-*, and *Dyokappappillomavirus*, have been identified from 28 different types of Papillomavirus, causing various pathological outcomes [1]. Multiple BPV types are found in each genus; *Delta papillomavirus* has 4 species (BPV-1, BPV-2, BPV-13, and BPV-14), *Xi Papillomavirus* (BPV-3, BPV-4, BPV-6, BPV-9, BPV-10, BPV-11, BPV-12, BPV-15, BPV-17, and BPV-23), *Epsilon papillomavirus 1* (BPV-5 and BPV-8), *Dyokappappillomavirus* genus (BPV-16 and BPV-18), and *Dyoxipapillomavirus 1* (BPV-7, BPV-19, and BPV-21); other seven BPV types, BPV-20–BPV-22 and BPV-24–BPV-27, belong to unclassified genera [2–4]. *Papillomaviruses* are species-specific carcinomas that typically develop as papillomas or warts on the skin and mucosal epithelia, but BPV-1, BPV-2, and BPV-13 can infect horses as well as cattle [5]. BPV risk is higher in calves and yearlings; however, all ages can be affected [6]. It potentially leads to financial losses in animal husbandry due to mastitis, decreased milk and carcass output, and impaired hide quality [7].

Proliferative lesions are small to large nodular cauliflower warts, typically gray to black in appearance and rough and filiform [8]. BPV infect the body through wounds or injuries and spreads between animals through rubbing on infected items, grooming tools, earmarks, or milking contaminated milk [9], and venereal warts can be transferred sexually [10]. In addition, immunodeficient animals, malnourishment, genetic anomalies, and prolonged sun exposure increase the risk of papillomavirus infection [11].

Histological examination can identify epidermal pathogenic changes in bovine papilloma, often diagnosed clinically when the epidermis presents well-described warts or nodular lesions [12]. The polymerase chain reaction (PCR) technique is a crucial diagnostic tool used to confirm infection by identifying specific BPV DNA [13]. Furthermore, sequencing and phylogenetic analysis were utilized to validate the results, as they are the most effective methods for characterizing BPV [14]. The prevalence of BPV in Egypt is under-researched, with some previous studies containing limited clinical and epidemiological studies [15]. Thus, the study aimed to genetically characterize BPV-1 circulation in clinically suspected BPV-infected cattle in Al-Sharkia, Egypt, using histology and PCR assays, and conduct further phylogenetic analysis to determine the isolates' relationships to other isolates in Egypt and around the world.

## 2. Materials and Methods

### 2.1. Animals and Sample Collection

This research was conducted in the summer of 2024, involving 27 crossbred cattle with suspected cutaneous papillomatous lesions. The study took place in two separate locations within the Al-Sharkia governorate of Egypt. First, there is the veterinary clinic at Zagzig University (*n* = 11), and second, there is a private clinic (*n* = 16) in Abu Hammad City, which is located approximately 17 km southeast of Zagazig, the seat of the governorate. These animals are raised by small-scale breeders who have limited knowledge of hygiene practices, injury prevention, and proper nutrition. Cattle diagnosed up to 2 years old with no apparent infection symptoms in their medical histories. Clinical examination focusing on the vital parameters including body temperature, heart, and respiratory rates was done for all examined animals. During clinical examination, animals appeared healthy except for wart-like lesions in various parts of the body as the neck, shoulders, legs, abdomen, scrotum, teats, and udder. Tissue specimens were obtained from each diseased animal during a routine clinical examination to confirm the diagnosis and obtain additional information. Samples were divided into two sections: one frozen for molecular diagnosis via PCR technique and the other fixed in 10% neutral buffered formalin for histological analysis. The samples were transported to the diagnostic laboratory under cold chain conditions (4°C) using insulated containers with ice packs to preserve sample integrity and prevent degradation.

### 2.2. Histopathological Examination

Skin tumor mass tissue samples were fixed for 24 h in neutrally buffered formalin 10%, then dehydrated in ethanol concentrations ranging from 70% to 100%, washed in xylene, and embedded in paraffin wax. An automated microtome was used to cut paraffin slices that were 5 μm thick. Standard Hematoxylin and Eosin (H&E) were then used to stain the slices [16].

### 2.3. PCR

The samples were minced with sterile scissors and forceps on a Petri dish. About 25 mg of tissue was weighed, washed in PBS, and transferred into 1.5 mL Eppendorf tubes. DNA was extracted using the QIAamp DNA Mini kit, with modifications made according to the manufacturer's instructions. In brief, 200 μL of the sample suspension was incubated with 20 μL of proteinase K and 200 μL of lysis buffer at 56OC for 10 min. After incubation, 200 μL of 100% ethanol was added to the lysate. The sample was then washed and centrifuged following the manufacturer's recommendations. Nucleic acid was eluted with 100 μL of elution buffer provided in the kit. Primers used were supplied from Metabion (Germany) are listed in Table 1. Primers were utilized in a 25 μL reaction containing 12.5 μL of EmeraldAmp Max PCR Master Mix (Takara, Japan), 1 μL of each primer of 20 pmol concentration, 5.5 μL of water, and 5 μL of DNA template. The reaction was performed in an Applied biosystem 2720 thermal cycler according to the condition listed in Table 1. PCR products were separated on a 1.5% agarose gel at 5 V/cm, with 20 μL of each sample loaded per well. DNA fragment sizes were determined using a 100 bp ladder (Fermentas, Thermo Fisher, Germany). The gels were imaged using a gel documentation system (Alpha Innotech, Biometra), and band analysis was performed using ImageJ software.

### 2.4. Sequencing and Phylogenetic Analysis

The purification procedure outlined in the QIAquick PCR Product Extraction Kit was employed to refine the amplified PCR products. Sequencing was carried out in both directions utilizing an Applied Biosystems 3130 genetic analyzer in conjunction with the BigDye Terminator V3.1 cycle sequencing kit from Perkin-Elmer (HITACHI, Japan). The sequences were analyzed using NCBI-BLAST after PCR results of three positive samples with the highest band intensity were received [18] to verify the identity of the sequences about GenBank accessions. The MegAlign module of Lasergene DNA Star version 12.1 [19] carried out the phylogenetic analysis, and the MEGA program Version 6.0 software [20] was used to generate the phylogenetic tree using the maximum likelihood, neighbor-joining approach.

## 3. Results

### 3.1. Clinical Examination

**Figure 1:**
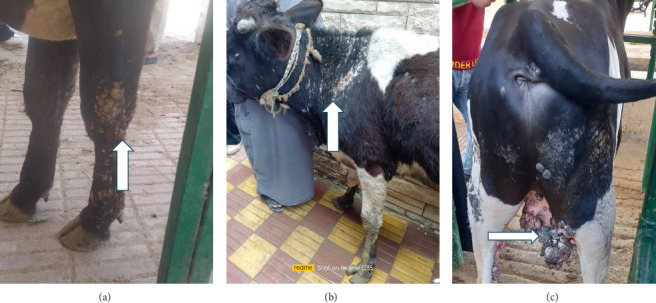
Gross pictures of cattle affected with cutaneous papilloma.

The results of the vital parameters were within the normal range for body temperature (38°C–38.5°C), heart rate (55–80/m), and respiratory rate (10–30/m) in all examined animals. Most cases had a widespread distribution of skin warts over various body parts, such as the head, neck, chest, belly, and legs (Figure 1).

### 3.2. Detection of BPV-1 Using Conventional PCR

**Figure 2:**
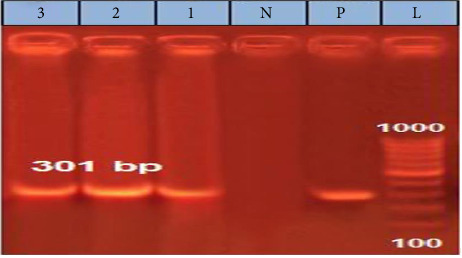
The specific product of BPV-1 in bovine wart samples. L: 100 bp DNA Ladder, 1–3: specific 301 bp band representing positive samples, N: negative control, and P: positive sample.

A partial amplification of the expected ∼301 bp band from the L1 gene was successfully observed in all analyzed samples, with three representative samples shown in the gel in the following (Figure 2).

### 3.3. Sequencing and Phylogenetic Characterization

**Figure 3:**
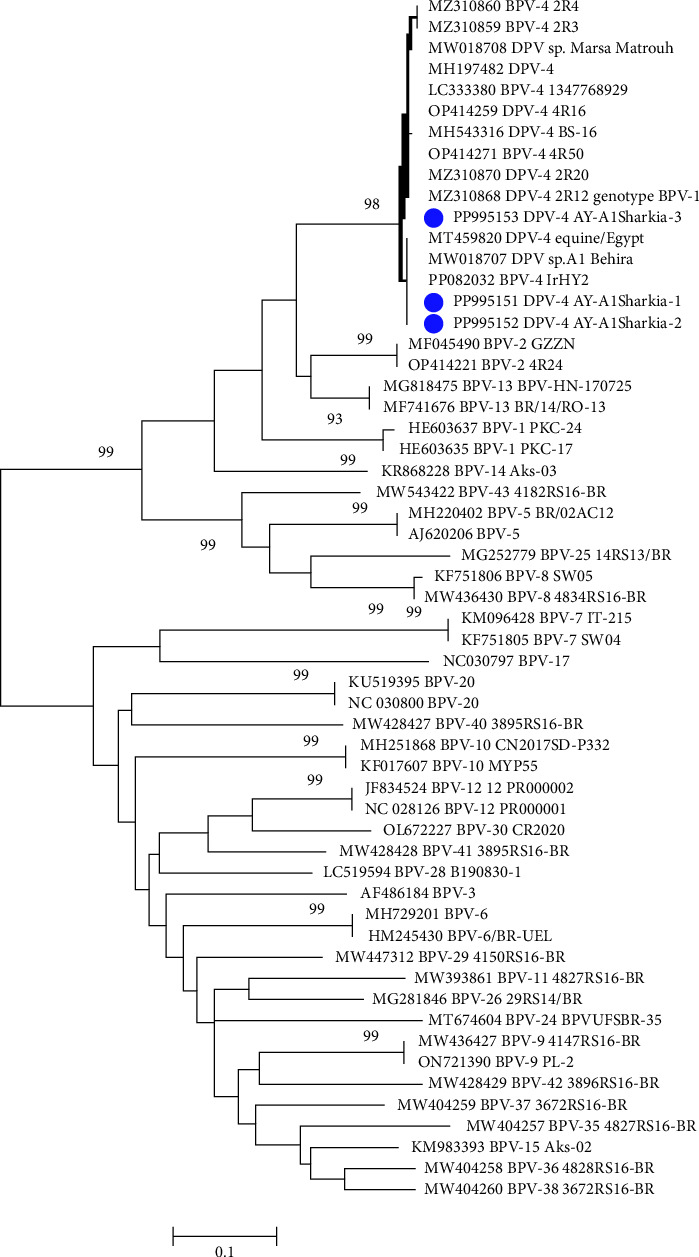
The phylogenetic tree was calculated with MEGA 6 software using the maximum likelihood method.

The partial nucleotide sequence analysis of three representative samples revealed the identification of only one type (Deltapapillomavirus 4; isolates; BPV-1 AY-AlSharkia) with the accession numbers PP995151, PP995152, and PP995153. Two isolates, PP995151 and PP995152, were 100% identical to each other at the nucleotide level while showing 98.9% homology with the PP995153 strain. Our sequence data and the phylogenetic analysis (Figure 3) clustered the Egyptian BPV of this study, PP995151 and PP995152, in the same clade with 100% nucleotide homology with sequences previously reported in Egypt including MW018707 and MT459820 from equine skin warts, as well as bovine sequences from Iraq (PP082038). Moreover, our three sequences were 98.6%-99.6% identical to sequences isolated from calves in Japan (LC333380), cattle in Turkey (MH197482), and sequences from Belgium detected from cattle(OP414271) and equine (OP414270 and MZ310868).

### 3.4. Histopathological Findings

**Figure 4:**
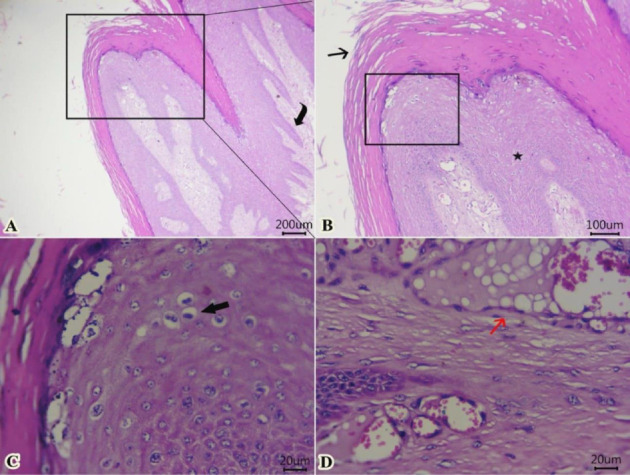
Representative photomicrographs of H&E-stained sections from tumor mass of skin showing (A–D) benign neoplasm with hyperkeratosis (arrow) and acanthosis (star) with finger-like projections toward the dermal layer (curved arrow). The proliferated epithelium formed from well-organized mature epithelial cells with “Koilocytes” (thick arrow). The fibrovascular core within the dermal layer contains dilated blood vessels with intravascular fatty vacuoles (red arrow) (Scale bar: 200, 100, and 20 μm, respectively).

Examined sections from wart lesions (Figure 4) showed benign neoplasm with hyperkeratosis (thickening of stratum corneum) and acanthosis (thickening of the stratum spinosum) with finger-like projections toward the dermal layer. The proliferated epithelium formed from well-organized mature epithelial cells with the presence of “Koilocytes” (abnormally shaped squamous epithelial cells with perinuclear cytoplasmic halo zone, wrinkled nuclear membrane, and hyperchromasia). In addition, fibrovascular cores were seen within a dermal layer and covered by exophytic squamous proliferation. Some dilated venous or arteries within the dermal layer contain intravascular fatty vacuoles. The presence of “Koilocytes” within proliferated epithelium could be attributed to the accumulation of viral proteins of “papillomavirus” and other cellular debris within the perinuclear region of koilocytes.

**Figure 5:**
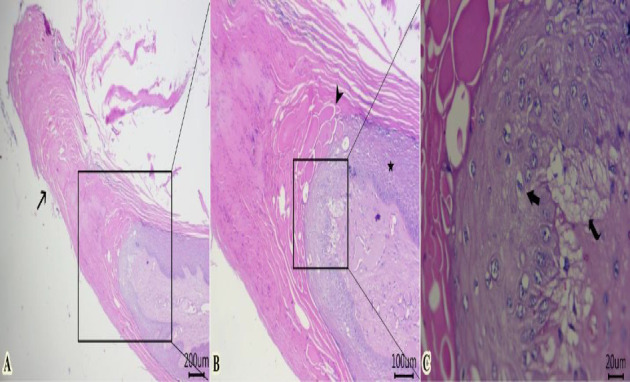
Representative photomicrographs of H&E-stained sections from tumor mass of skin showing (A) papillary projections formed from marked elongation of hyperkeratosis (arrow). (B) Islets or globules of hyperkeratosis (arrowhead) particularly at the surface of stratum lucidum. (C) Proliferated stratum spinousm with koilocyte (thick arrow) and subepidermal vacuolation (curved arrow) (Scale bar: 200, 100, and 20 μm, respectively).

Some examined areas of tumor mass revealed papillary projections formed from marked elongation of hyperkeratosis (Figure 5(A)). The latter was formed from islets or globules, particularly at the surface of stratum lucidum (Figure 5(B)). Proliferated stratum spinous with koilocytes, and subepidermal vacuolation were also encountered (Figure 5(C)).

## 4. Discussion

BPVs are oncogenic DNA viruses that infect mammals, causing an infectious disease that spreads globally and results in substantial economic losses [12]. BPV causes clinical issues such as cattle hide damage, body condition loss, and teat and udder infections, impacting animal sales, production, and dairy industries [2]. There is a lack of molecular data about the circulating strains, their genetic relatedness, and their origins in Egypt [21]. This study aimed to identify and molecularly characterize the virus genotype in the Al-Sharkia governorate using phylogenetic analysis using local and global BPV to detect evolutionary changes.

Our study revealed long-lasting, variably sized, circumscribed warts in clinical cases of bovine papillomatosis distributed across various body regions—including teats, as well as haired and hairless areas. These findings are consistent with earlier studies [22, 23] but contrast with reports such as Vitiello et al. [24], who observed lesions localized only to the teats of heifers. The study found that all infected cattle aged 6–24 months, the same age group found in previous research by [12, 25], which demonstrates the age-related infection and the higher susceptibility of young ages to infection. Age-related infection in young calves is higher due to ill-developed immune systems, stress factors like weaning, and parasitic infestations, which may lower their immunity and facilitate virus infection.

Conventional PCR targeting the L1 gene of BPV-1 using specific primers revealed the detection of BPV-1 in all 27 examined wart samples. This finding is aligned with previous research which reported BPV-1 as the most detected type from cattle aged less than 2 years revealing such clinical signs in different countries including Egypt [15, 26]. The study is the first to report BPV-1 infection in cattle under 2 years old in Al-Sharkia governorate. The obtained sequences in this study of partial L1 nucleotide fragments from three representative samples were 98%–100% homologous to the *Deltapapillomavirus* 4 isolates and were submitted in the GenBank under the accession numbers PP995151, PP995152, and PP995153. Therefore, these obtained sequences were considered to belong to only one type (Deltapapillomavirus 4) according to the general rules applied in PV taxonomy, where a nucleotide sequence variance of more than 10% in the L1 gene with the closest strain is needed to identify a new genotype [27]. The present findings are further supported by a growing body of literature that highlights the global diversity and distribution of bovine papillomavirus types. Notably, Zhu et al. [28] reported the first detection of BPV-10 in dairy cattle in China. Complementing this, Savini et al. [29] identified the presence of BPV types 6, 7, 8, 10, and 12 in cattle from Italian herds. More recently, Yildirim et al. [28] characterized isolates from bovine teat tissues in Turkey, identifying three as BPV-6, two as BPV-10, one as BPV-2, and one as BPV-8. Similarly, Kale et al. [30] analyzed lesions from 20 cattle with teat papillomatosis randomly sampled across different herds in Turkey. Through phylogenetic analysis, they confirmed three isolates as BPV-1 (Deltapapillomavirus 4) and one as BPV-2, further demonstrating the coexistence of multiple BPV types in clinical lesions. These studies support the use of the L1 gene for BPV detection and confirm the presence of multiple BPV types in cattle lesions, emphasizing the complexity of papillomavirus infections globally.

Interestingly, two isolates of the present study, PP995151 and PP995152, were clustered in the same clade with equine Delta papillomavirus 4 (DPV4) sequences detected in Egypt including MW018707 from Al Behira in 2019, MT459820 in 2020, with 100% nucleotide identity and MW018708 from Marsa Matrouh in 2019 with 98.6% homology at nucleotide similarity. The third isolate, PP995151, in this study, was clustered together with equine isolates reported in Belgium (MZ310859 and MZ310868). Recent studies have reported the common occurrence of BPV-1 cross-species transmission, with BPV-1 being highly reported infecting equine and bovine hosts [31]. The authors in [32] stated that cross-species transmission between cattle and horses is expected to be an increasing process, with equine and bovine hosts sharing similar nucleotide polymorphismss and equine-derived BPV-1 mutations not yielding horse-adapted variants. Moreover, the three isolates of the present study revealed 98.2%–100% nucleotide homology with bovine sequences from New Valley, Egypt (MH543316), Iraq (PP082038), Turkey (MH197482), and Belgium (OP414271 and OP414259). By comparing our BPV-1 sequences with global strains, including those from Iraq, Turkey, Belgium, and China, we observed strong genetic similarities, supporting the hypothesis of cross-border transmission potentially linked to cattle trade. These findings reinforce the relevance of our study beyond Egypt and suggest that our data contribute meaningfully to the global understanding of BPV epidemiology and phylogeny.

This study found varying cellular proliferation in both epidermal and dermal layers, acanthosis with finger-like papillae, and benign exophytic mass formation of epithelia with papillomatosis, consistent with previous findings [33, 34]. Furthermore, we included observations of koilocytosis, hypergranulosis, and hyperkeratosis, which are characteristic of papillomavirus-induced lesions. Koilocytosis is a hallmark cytopathic effect of papillomavirus, resulting from the accumulation of viral proteins that disrupt normal cellular architecture and function. Earlier reports suggest that the interaction of oncogenic protein E5 with various proteins can lead to cell loss of adhesiveness and cell polarity [35, 36]. Moreover, the presence of fibrovascular cores in the dermal papillae further supports the diagnosis of papillomatosis. These findings reflect active epithelial proliferation and viral cytopathic effects. Also, the formation of keratin islets or globules, especially at the stratum lucidum, and the proliferation of the stratum spinosum with associated koilocytes, further support a diagnosis involving abnormal keratinocyte differentiation and viral cytopathic effects. The virus's assembly within proliferated cells led to an increase in the production of keratin granules, which serve as physical protection for the virus [37].

## 5. Conclusion

Egypt's BPV studies primarily focus on electron microscopy and histopathological examination, leaving a gap in understanding circulating strains, their genetic relationships, and their origins. In this study, the L1 gene of BPV-1, the most detected type from cattle aged less than two years worldwide, was detected in all 27 examined wart samples. Of interest was the study that found three isolates, highly similar to equine DPV4 sequences, indicating cross-species transmission of BPV-1 between equine and bovine hosts. Moreover, the three isolates of the present study revealed 98.2%–100% nucleotide homology with bovine sequences from Egypt, Iraq, Turkey, and Belgium. Therefore, the potential transmission of BPV between countries during animal trade requires further attention. Also, further molecular studies on genetic diversity among BPVs in Egypt could aid in identifying other types and provide information on their origin, transmission, and genetic relatedness.

## Figures and Tables

**Table 1:** Primers sequences, target genes, amplicon sizes, and cycling conditions.

Target gene	Primers sequences	Amplified segment (bp)	Primary denaturation	Amplification (35 cycles)			Final extension	Reference
				Secondary denaturation	Annealing	Extension		
Bovine papilloma virus L1 gene	GGAGCGCCTGCTAACTATAGGA	301	94°C 5 min	94°C 30 s	60°C 40 s	72°C 40 s	72°C 10 min	[17]
	ATCTGTTGTTTGGGTGGTGAC							

## Data Availability

The data that support the findings of this study are available from the corresponding author upon reasonable request.
